# The association between adherence to antiretroviral therapy and viral suppression under dolutegravir‐based regimens: an observational cohort study from Uganda

**DOI:** 10.1002/jia2.26350

**Published:** 2024-08-18

**Authors:** Zachary Wagner, Zetianyu Wang, Chad Stecher, Yvonne Karamagi, Mary Odiit, Jessica E. Haberer, Sebastian Linnemayr

**Affiliations:** ^1^ Center for Economic and Social Research University of Southern California Los Angeles California USA; ^2^ Department of Economics Sociology and Statistics RAND Corporation Santa Monica California USA; ^3^ Pardee RAND Graduate School Santa Monica California USA; ^4^ College of Health Solutions Arizona State University Phoenix Arizona USA; ^5^ Mildmay Uganda Kampala Uganda; ^6^ Massachusetts General Hospital Boston Massachusetts USA; ^7^ Harvard Medical School Boston Massachusetts USA

**Keywords:** antiretroviral therapy, dolutegravir, HIV, medication adherence, Uganda, viral suppression

## Abstract

**Introduction:**

Millions of people living with HIV (PLWH) take oral antiretroviral therapy (ART), which requires a lifetime of consistent medication adherence. The relationship between adherence and poor HIV outcomes is well documented. Newer ART regimens that include dolutegravir (DTG) could be more forgiving, but empirical evidence on the relationship between adherence and viral suppression under DTG is only emerging.

**Methods:**

In this observational cohort study (secondary analysis of data from a randomized trial), we used data from 313 ART clients from a large HIV clinic in Kampala, Uganda. Over the 4‐year study period (January 2018–January 2022), 91% switched from non‐DTG regimens to DTG regimens. We measured adherence using Medication Event Monitoring Systems‐caps and extracted prescription information and viral load measures from electronic health records. We estimated unadjusted linear regressions and adjusted models that included individual and time fixed‐effects.

**Results:**

Under non‐DTG regimens, 96% of participants were virally suppressed (defined as viral load < 200 copies/ml) when adherence was 90% or higher in the 3 months before viral load measurement. Viral suppression was 32 percentage points lower when adherence was between 0% and 49% (95% CI −0.44, −0.20, *p* < 0.01), 12 percentage points lower when adherence was between 50% and 79% (95% CI −0.23, −0.02, *p* < 0.01), and not significantly different when adherence was between 80% and 89% (effect of 0.00, 95% CI −0.06, 0.07, *p* = 0.81). In contrast, for participants taking DTG, there was no statistically significant difference in viral suppression among any of the four adherence levels; more than 95% were virally suppressed at each adherence level. On average, switching to DTG increased viral suppression by 6 percentage points in our adjusted models (95% CI 0.00, 0.13, *p* = 0.03).

**Conclusions:**

There was no significant association between adherence levels and viral suppression among PLWH taking DTG regimens, suggesting a high degree of forgiveness for missed doses. The use of DTG should be prioritized over older regimens, particularly for those with low adherence.

**Clinical Trial Number:**

NCT03494777.

## 1 INTRODUCTION

Antiretroviral treatment (ART) for HIV is one of the most important medical advances of the modern era. This innovation has saved millions of lives and transformed an illness that was once thought of as a death sentence into a manageable chronic condition [1, 2]. Today, one key objective of HIV researchers and practitioners is to ensure that all people living with HIV (PLWH) start ART as soon as possible and persist with lifelong adherence to sustain viral suppression [3]. However, maintaining high levels of ART adherence is challenging, and many PLWH fail to adhere at optimal levels [4]. In low‐resource settings, structural challenges, such as ART supply shortages and limited access to clinics for refills, as well as behaviour challenges such as treatment fatigue, can hinder the ability to adhere. As a result, many interventions are being tested and implemented in low‐income settings that try to improve ART adherence [5, 6, 7, 8, 9, 10, 11].

The strong relationship between high ART adherence and viral suppression has been well documented with a variety of ART drug classes [12, 13, 14, 15, 16]. However, newer ART drug regimens that include dolutegravir (DTG), an integrase strand transfer inhibitor (INSTI), are thought to be more forgiving because of higher potency [17] and a higher barrier to resistance, particularly in second‐generation INSTIs like DTG and bictegravir [18, 19]; missing doses may, therefore, have less of an effect on viral suppression compared to non‐DTG regimens [17, 20, 21]. However, some studies have found that higher adherence to DTG‐based regimens is associated with a higher likelihood of achieving viral suppression compared to lower adherence levels [22, 23, 24, 25, 26]. Understanding the importance of adherence for suppression under DTG‐based regimens is important because encouraging adherence behaviour that is overly stringent can demotivate ART patients [27, 28] and negative attitudes from providers about low adherence can lead to loss from care [29]. As more and more people shift to DTG [30], clinical guidance and counselling messages could greatly benefit from estimates of what levels and patterns of adherence are necessary to achieve viral suppression under these new regimens and to what extent switching to DTG improves viral suppression.

In this study, we use longitudinal electronically measured adherence data linked to viral load test results to estimate the relationship between adherence and viral suppression in Uganda. Consistent with many parts of sub‐Saharan Africa, 91% of participants switched from non‐DTG ART regimens to DTG over our 4‐year study period (January 2018–January 2022). This transition allows us to estimate the association between ART adherence and viral suppression before and after switching to DTG for the same people, and to estimate the effect of switching to DTG on viral suppression. We focus on three key questions that will improve our understanding of how ART adherence impacts viral suppression for patients on DTG: (1) What is the relationship between mean adherence (the share of prescribed doses that were taken) and viral suppression under DTG and how does this compare to non‐DTG regimens? (2) How much do sustained adherence interruptions affect viral suppression under DTG? (3) How does switching from non‐DTG to DTG affect viral suppression on average?

## 2 METHODS

### 2.1 Setting and sample

Our study data are from Mildmay Uganda, one of the largest and most experienced HIV clinics in the country. Services at Mildmay include free HIV counselling and testing; paediatric and adult HIV prevention, treatment and care services; diagnostic (laboratory) services and radiology. The Mildmay Uganda laboratory is accredited by the South African National Accreditation System for International Origination for Standardization for virologic assessment. Of the 15,000 patients served at Mildmay Uganda, 11% are children below 18 years, 65% are female and 100% of all clients in care take ART. Mildmay is one of a growing number of facilities with a well‐established electronic health record (EHR) system in Uganda. Mildmay Uganda started slowly rolling out DTG midway through 2018 and our study period includes January 2018−January 2022.

The sample used for this study were all enrolled in a randomized controlled trial (“Behavioral Economics Incentives to Support HIV Treatment Adherence” or BEST, NCT03494777). The BEST study tested an intervention that promotes ART adherence through small rewards for high adherence and is described in detail elsewhere [6]. The intervention did not significantly impact adherence or viral suppression, and thus it is unlikely to confound the results of the current study [31]. Enrolment criteria for BEST (and subsequently this study) included being 18 years of age or older, receiving ART for 2 or more years, reported as having recent adherence problems in the EHR, speaking either English or Luganda (the local language spoken by most people in and around Kampala) and willingness to use an electronic pill cap (Medical Event Monitoring System [MEMS]) to record adherence. Exclusion criteria included unwillingness or inability to regularly use the MEMS cap. Eligible clients were identified prospectively using the EHR database. We selected a random sample from all eligible clients prospectively from the EHR records and enrolled them during their next clinic visit. This avoids potential disadvantages of convenience sampling, which could lead to enrolment of only clients who were easiest to contact or who come into the clinic more frequently. For the current study, we followed participants for an average of 36 months (range of 4.5−44.5) depending on when they were enrolled. The transition to DTG started roughly midway through the study and participants were switched based on Uganda Ministry of Health Guidelines, which required a viral load of <1000 c/ml with no contraindications. Participants eligible for DTG‐based regimens were switched in a staged process as clients presented for routine care.

### 2.2 Study data

#### 2.2.1 Adherence

Participants in the BEST study all received a MEMS‐cap (made by AARDEX) upon recruitment to store their medication. MEMS‐caps house a microelectronic chip that records the date and time of each bottle opening, enabling an objective assessment of the timing of each dose removal (as a proxy for each dose taken). We used the doses listed in the client's pharmacy refill record for calculation of adherence, and updated this information when there was a regimen change. We had clients put the MEMS‐cap on one medication bottle and used the one that required the most frequent dosing. We used the MEMS‐cap event data to create a client‐day dataset that records the client's adherence for each day that they had the device. Lost devices were replaced, and difficulties using the device were discussed with the participant in such cases to avoid future loss or non‐use when prolonged periods of no device openings were detected. We created a daily adherence variable that captures the share of prescribed pills that were taken each day by dividing the number of bottle openings on each day by the number of doses prescribed (capped at 100%).

#### 2.2.2 HIV viral load

We extracted data on HIV viral load measures from the EHR. We used these data to create our binary outcome variable, an indicator for whether the client has a suppressed viral load, which we defined as below 200 copies/ml [32]. We also tested sensitivity to coding suppression as below 50 copies/ml. In our study period, clients had between 2 and 7 viral load tests.

#### 2.2.3 Linking adherence and viral suppression

For each viral load test, we combined the adherence data for the 3 months leading up to the viral load test date and tested sensitivity to using 1‐ and 6‐month look‐back period. Thus, each viral load test was a separate row in our data set with corresponding adherence information. We calculated the 1/3/6‐month average adherence (share of doses taken) in the period proceeding each test by taking the average daily adherence over the 30/60/90 days leading up to the test and categorized the measure into four groups: 0%–49%, 50%–79%, 80%–89% and 90%–100%, with the last group as the reference group throughout the analyses. We also identified whether participants had consecutive‐day gaps in adherence (1 day, 2 days, 3–5 days and 6+ days) during the period leading up to the viral load test.

### 2.3 Empirical analysis

To investigate the relationship between adherence and viral suppression under each of the two regimens (DTG and non‐DTG), we used ordinary least squares (OLS) regression models with interaction terms. Specifically, we estimated an unadjusted model where we regressed whether a test was virally suppressed (binary outcome) on the average 3‐month adherence level (four categories, with “90%−100%” being the reference), an indicator for whether the participant switched to DTG prior to their test (binary outcome), and interaction terms between adherence and the DTG indicator. We present associations between adherence and suppression for non‐DTG using the stand‐alone coefficients on each adherence bin and for DTG using the interaction terms added to stand‐alone coefficients. We also estimated an adjusted model where we control for individual and time fixed‐effects (equivalent to adding dummy variables for each individual and dummy variables for each quarter in the study period). Our fixed‐effects models estimate relationships within the same individuals over time. This controls for selection bias, and any client characteristics that do not change over time. Thus, things like the history of virological failure, when ART was initiated and pre‐existing comorbidities cannot confound our estimates. We clustered standard errors by individual in all analyses. We used linear probability models instead of logistic regression models because of well‐documented challenges with interpreting interaction effects in logistic regression [33] and potential issues such as the incidental parameter problem with fixed‐effects in logistic regression [34]. However, we also estimated logistic regressions to test the sensitivity of our results to the choice of modelling approach (Supplementary Appendix Table A14).

To investigate whether having consecutive‐day gaps with zero pills taken was associated with a lower likelihood of viral suppression, we regressed whether a test was virally suppressed on binary indicators for whether there was a 1‐day adherence gap, 2‐day gap, 3‐ to 5‐day gap, 6+ day gap, an indicator for DTG, and interaction terms with the DTG indicator and each of the binary gap indicators. Unadjusted and adjusted models were estimated in the same fashion described above.

To estimate the effect of switching to DTG on viral suppression, we used an OLS model with individual and quarter (3‐month interval) fixed‐effects. This model compares the probability of suppression from before to after switching to DTG for the same individual, controlling for trends in suppression.

With 664 viral load test observations for participants prior to switching to DTG, we can detect significant differences in viral suppression between the four adherence groups of about 7.4 percentage points with a power of 0.8. With 367 viral load test observations after switching to DTG, we can detect significant differences in viral suppression between the four adherence groups of about 9 percentage points.

### 2.4 Ethical considerations

We obtained ethics approval from the RAND Corporation's Human Subjects Protection Committee (#2016‐0956), the Mildmay Uganda Research Ethics Committee Institutional Review Board (#02013‐2018) and the Uganda National Council for Science and Technology (#2394). All participants provided written informed consent.

## 3 RESULTS

Between 12th April 2018 and 14th May 2019, we approached 432 pre‐identified clients based on the eligibility criteria and recruited 375 clients for potential study participation (35 refused to participate and 22 turned out to be ineligible). Of those recruited, 46 clients dropped out or were deemed ineligible prior to enrolment. Lastly, we dropped 16 participants who did not have at least two viral load tests, leading to a final count of 313 participants and 1031 viral load tests in our study. Table 1 shows that participants were 37 years old on average and 35% were male; 59% of participants could read and 55% completed secondary school; participants had an average monthly income of about $58 USD per month, 63% were employed and 21% were food insecure; the average travel time to the clinic was 2 hours. Almost all (93%) of participants entered the study on a non‐DTG ART regimen and 91% of those switched to DTG at some point during the study period. The most common non‐DTG regimens were Tenofovir/Lamivudine/Efavirenz (29%) and Atazanavir/Ritonvir + Tenofovir/Lamivudine (27%) and the most common DTG‐based regime was Tenofovir/Lamivudine/Dolutagravir (82%; see Supplementary Appendix Table S13 for full list of DTG and non‐DTG based regimens). Figure S1 shows the distribution of adherence under DTG (IQR 57%−98%) and non‐DTG regiments (IQR 57%−97%). Of all 1031 tests included in the sample, 866 (84%) showed a viral load of less than 200 copies/ml.

**Table 1:** Summary of participant characteristics at baseline

Number of participants	313	
Number of viral load tests	1031	

	Mean	SE
Number of tests per participant	3.29	0.05
Share with viral load < 200 copies/ml (%)	84.0	1.13
**Demographics**		
Age (years)	37.4	0.730
Male (%)	35.4	2.70
English preferred language (%)	31.9	2.63
Literate (%)	58.7	2.78
Completed secondary school (%)	54.6	2.81
In a relationship (%)	51.1	2.82
**Poverty**		
Monthly income (USD)	57.8	4.97
Employed (%)	63.2	2.72
Food insecure (%)	21.4	2.32
**Structural**		
Time to reach clinic (hours)	2.01	0.078
**Stigma**		
Comfortable disclosing status (%)	51.7	2.82

*Note*: All summary statistics were calculated at the participant level. Monthly income was converted to USD using a conversion factor of 1 USD = 3400 Ugandan Shilling. Food insecurity was measured using the Food Insecurity Experience Scale and we defined food insecure as a raw score of 4 or 5 as has been done in prior studies [35].

### 3.1 Association between adherence and viral suppression under different regimens

**Figure 1:**
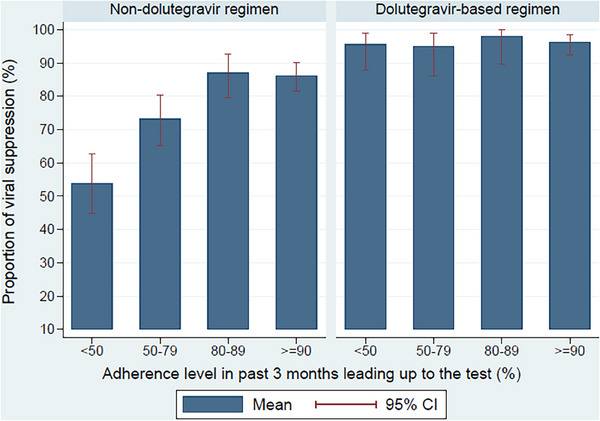
Share of participants with a suppressed viral load under different levels of adherence by DTG regimens. Analyses were run at the viral load test level and include 313 individuals and 1031 viral load tests. Sample sizes for each non‐DTG adherence group are: <50 = 130, 50–79 = 142, 80–89 = 116 and ≥90 = 276. Sample sizes for DTG adherence groups are: <50 = 69, 50–79 = 60, 80–89 = 51 and ≥90 = 187.

Figure 1 shows that when participants were on non‐DTG regimens, there was a clear negative association between adherence and viral suppression: more than 85% of those with adherence above 80% were suppressed compared to only 52% when adherence was less than 50%. However, when on DTG, there was no relationship between adherence and suppression; more than 95% were suppressed at all adherence levels.

Table 2 shows regression results assessing the relationship between viral suppression and adherence under different regimens. Compared to 90% adherence or above, the probability of viral suppression with non‐DTG regimens was 32 percentage points lower when adherence was between 0% and 49% (95% CI −0.44, −0.20, *p*<0.01), 12 percentage points lower when adherence was between 50% and 79% (95% CI −0.23, −0.02, *p*<0.01), and not significantly different when adherence was between 80% and 89% (95% CI −0.06, 0.07, *p* = 0.81). In contrast, when participants were taking DTG, there was no statistically significant difference in viral suppression between the four adherence levels. Adjusted models that included patient and quarter fixed‐effects show similar conclusions.

**Table 2:** Adherence and viral suppression under dolutegravir versus non‐dolutegravir ART regimens

Adherence level: relative to >90%	Association with viral suppression							
	non‐dolutegravir regimen				dolutegravir regimen			
	Unadjusted (95% CI)	*p*‐value	Adjusted (95% CI)	*p*‐value	Unadjusted (95% CI)	*p*‐value	Adjusted (95% CI)	*p*‐value
0%−49%	−0.32 (−0.44, −0.20)	0.00	−0.16 (−0.30, −0.02)	0.02	−0.00 (−0.06, 0.04)	0.82	0.05 (−0.04, 0.14)	0.26
50%−79%	−0.12 (−0.23, −0.02)	0.01	−0.11 (−0.24, 0.00)	0.06	−0.01 (−0.08, 0.06)	0.73	0.06 (−0.01, 0.15)	0.11
80%−89%	0.00 (−0.06, 0.07)	0.81	0.02 (−0.04, 0.10)	0.43	0.01 (−0.02, 0.06)	0.45	0.00 (−0.08, 0.08)	0.97

*Note*: Analyses were run at viral load test level (*n* = 1031) because most individuals had more than one viral load test. The table includes estimates from one unadjusted model and one adjusted model, both of which include interaction terms between dolutegravir (DTG) and adherence bins. Estimates for non‐DTG are from stand‐alone coefficients on each adherence bin. Estimates for DTG are stand‐alone coefficients on each bin added to the DTG interaction term for each bin. Adjusted models include individual fixed‐effects and quarter fixed‐effects (number of quarters = 15). Both unadjusted and adjusted models include 313 individuals. 95% CIs and *p*‐values were estimated with standard errors clustered by individual.

Table 3 shows that consecutive‐day interruptions in adherence reduced the probability of suppression when on non‐DTG regimens but not when on DTG. When on non‐DTG regimens, experiencing any interruption of 6 consecutive days or longer was associated with a 28 percentage points reduction in suppression (95% CI −0.37, −0.18, *p*<0.01), controlling for average adherence level. In contrast, when on DTG, having any interruption of 6+ consecutive days had no significant effect on adherence (95% CI −0.03, 0.08, *p* = 0.48). Results show similar overall conclusions in adjusted models.

**Table 3:** Adherence interruptions and viral suppression under DTG versus non‐DTG ART

Duration of interruption	Difference in viral suppression relative to no gaps							
	non‐dolutegravir regimen				dolutegravir regimen			
	Unadjusted (95% CI)	*p*‐value	Adjusted (95% CI)	*p*‐value	Unadjusted (95% CI)	*p*‐value	Adjusted (95% CI)	*p*‐value
1 day	−0.03 (−0.11, 0.05)	0.47	−0.03 (−0.11, 0.05)	0.44	0.02 (−0.03, 0.09)	0.37	0.05 (−0.02, 0.13)	0.14
2 days	−0.07 (−0.16, 0.01)	0.10	−0.03 (−0.12, 0.04)	0.37	−0.02 (−0.07, 0.03)	0.43	−0.04 (−0.12, 0.03)	0.31
3−5 days	−0.02 (−0.09, 0.05)	0.57	−0.04 (−0.12, 0.03)	0.25	−0.04 (−0.12, 0.02)	0.23	0.00 (−0.08, 0.09)	0.95
6 days or more	−0.28 (−0.37, −0.18)	0.00	−0.14 (−0.23, −0.05)	0.00	0.02 (−0.03, 0.08)	0.48	0.01 (−0.06, 0.10)	0.68

*Note*: Analyses were run at viral load test level. The table includes estimates from one unadjusted model and one adjusted model, both of which include interaction terms between dolutegravir (DTG) and interruption duration bins. Estimates for non‐DTG are from stand‐alone coefficients on each duration bin. Estimates for DTG are stand‐alone coefficients on each bin added to the DTG interaction term for each bin. Adjusted models include individual fixed‐effects and quarter fixed‐effects (number of quarters = 15). Both unadjusted and adjusted models controlled for the average adherence level before each test. Both unadjusted and adjusted models include 313 individuals. 95% CIs and *p*‐values were estimated with standard errors clustered by individual. The duration of interruption categories is not mutually exclusive. Therefore, the coefficients shown above are net effects of experiencing adherence gap of certain length, controlling for the presence of gaps of all other lengths.

### 3.2 Association between switching to DTG and viral suppression

Table 4 shows that the share of participants with a suppressed viral load was 18 percentage points higher when participants were on DTG compared to non‐DTG regimens (95% CI 0.13, 0.23, *p*<0.01). Respondents on DTG had significantly higher levels of viral suppression at every level of adherence, but differences were larger at lower levels of adherence. Adjusted models that control for patient and quarter fixed‐effects show that switching to DTG increased suppression by 6 percentage points (95% CI 0.00, 0.13, *p* = 0.03). This effect is present despite the fact that switching to DGT reduced average adherence by 8 percentage points (95% CI −0.12, −0.04, *p*<0.01; Table S1, Supplementary Appendix).

**Table 4:** Association between DTG and viral suppression (VS)

	Difference in viral suppression for non‐dolutegravir regimen compared to dolutegravir regimens					
	Share of VS non‐dolutegravir	Share of VS dolutegravir	Unadjusted (95% CI)	*p*‐value	Adjusted (95% CI)	*p*‐value
**All levels of adherence pooled**	0.77 (0.74, 0.80)	0.96 (0.94, 0.98)	0.18 (0.13, 0.23)	0.00	0.06 (0.00, 0.13)	0.03
**Adherence level**						
0%−49%	0.53 (0.45, 0.62)	0.95 (0.90, 1.0)	0.41 (0.30, 0.52)	0.00	0.14 (−0.13, 0.42)	0.30
50%−79%	0.73 (0.65, 0.80)	0.95 (0.89, 1.0)	0.21 (0.10, 0.33)	0.00	0.20 (−0.20, 0.61)	0.31
80%−89%	0.87 (0.80, 0.93)	0.98 (0.94, 1.0)	0.10 (0.02, 0.19)	0.00	0.07 (−0.07, 0.22)	0.29
>90%	0.86	0.96	0.10	0.00	−0.00	0.80

*Note*: Analyses were run at viral load test level. Adjusted models include individual fixed‐effects and quarter fixed‐effects (number of quarters = 15). Both unadjusted and adjusted models include 313 individuals. 95% CIs and *p*‐values were estimated with standard errors clustered by individual.

### 3.3 Sensitivity analyses

Tables S2−S4 show similar overall conclusions when we used a 1‐month look‐back period for adherence, and Tables S5−S7 show that results are also similar when we used a 6‐month look‐back period. Tables S8−S10 show that results are robust to using 50 copies/ml to indicate viral suppression. Tables S11 and S12 show that results are also robust to controlling for viral suppression status in the previous test.

## 4 DISCUSSION

As the push to reach the UNAIDS targets of 95% of PLWH taking ART and 95% virally suppressed continues, it is critical to know how adherence relates to viral suppression with various regimens. This study shows that with DTG‐based regimens, high levels of suppression can be achieved even with relatively low ART adherence. In contrast, low adherence strongly reduced viral suppression under non‐DTG regimens. Moreover, switching to DTG increased viral suppression by 6 percentage points on average in our sample. Our results support the WHO's recommendation to make DTG the preferred first‐line treatment [30] and suggest that increasing uptake of DTG or bictegravir (i.e. second‐generation INSTIs) should be a key consideration for HIV care practitioners around the world.

We find higher rates of viral suppression with DTG versus non‐DTG regimens despite the fact that switching to DTG decreased adherence. Our results do not imply that people on DTG no longer need to strive for high ART adherence, but rather people with adherence problems are likely to fair better on DTG. Decreased adherence could be a problem in the long‐term for retention‐in‐care and ensuring continued utilization of other key HIV services. Decreased adherence could also lead to resistance to DTG in the long‐term despite its high genetic barrier relative to other regimens [36]. Our study does not include a long‐term follow‐up and these potential unintended consequences should be monitored.

Our findings add to the progression of adherence forgiveness that has been seen with increasingly potent and tolerable ART regimens over time. The initial goal of 95% adherence was established based on unboosted protease inhibitors [37], which have not been recommended for over a decade. Notably, participants in this study who were not taking DTG‐based regimens were largely receiving non‐nucleoside reverse transcriptase inhibitors (NNRTIs), and their viral suppression was highest at or above 80%. Moreover, the ability of interrupted adherence to maintain viral suppression with a variety of ART regimens (i.e. boosted Protease Inhibitors (PIs), NNRTI, and first‐ and second‐generation INSTIs) was recently demonstrated in the QUATUOR study—a randomized trial comparing daily ART to short‐cycle interruptions (i.e. 4 days on, 3 days off) [38]. The high rates of viral suppression with INSTIs are well established in the literature [22, 39] and indeed helped drive the rollout of DTG globally. Our analysis, however, is one of the first to use electronic adherence monitoring to show that potentially very low adherence with DTG‐based regimens can still result in viral suppression for most people.

Other studies have assessed the relationship between adherence and viral suppression with DTG. Notably, the NAMSAL and ADVANCE clinical trials—both conducted exclusively in sub‐Saharan Africa—demonstrated the non‐inferior efficacy of DTG‐based first‐line regimens compared to NNRTI‐based regimens for ART‐naïve individuals. NAMSAL did not find adherence to predict viral suppression based on mean medication possession ratios and self‐report categorized as (<80%, 80–95 and >95%) [39], similar to our findings. ADVANCE showed that self‐reported adherence <80% was predictive of viral suppression [40]. We believe the difference in this finding and our results may be due to differences in measurement and the well‐known potential for self‐reported adherence to be over‐estimated (e.g. due to social desirability bias) [41]. It is possible that those reporting <80% may have had very low adherence indeed. Similar positive associations between adherence and viral suppression were presented for another observational study in Ethiopia that used self‐report [24], as well as in the multinational GEMINI studies that used pill counts [23], which are also well‐known for potential over‐estimation of adherence. An Italian study did find the traditional positive linear relationship between adherence based on medication possession ratio and viral suppression. Differences with our findings may be due to the length of prior viral suppression and/or patterns of adherence (e.g. longer consecutive periods of non‐adherence).

Our study has several limitations. First, while electronically measured adherence has been shown to be a highly informative measure for adherence [42], it may not always reflect realized adherence. Second, this study has limited external validity as it only includes the treatment of mature clients from one hospital in Uganda. Results may differ with individuals initiating ART on DTG‐based regimens or in populations with different adherence patterns (e.g. more protracted consecutive non‐adherence). Third, there could be selection bias into DTG that our adjusted models do not account for. Fourth, we do not have long‐term follow‐up, so we are unable to assess whether the forgiving nature of DTG holds in the long‐run. Fifth, people could pocket doses of ART, in which case our MEMS‐cap measurement of adherence would be an underestimate (this would be similar for DTG and non‐DTG regimens). Sixth, the observational nature of our study raises the possibility of confounding; however, we controlled for confounders to the extent possible. Moreover, randomization to DTG is not feasible given the Uganda Ministry of Health's plan to switch all PLWH to DTG‐based regimens. Finally, the duration of viral suppression is a key factor in the ability to maintain viral suppression [43, 44] and results may differ in populations initiating DTG at different stages.

## 5 CONCLUSIONS

We found that when PLWH are on DTG‐based regimens, adherence to ART has much less of an association with viral suppression compared to when they are on non‐DTG‐based regimens. While further research is needed to corroborate these findings, they are encouraging for the prospects of meeting the UNAIDS targets. It is often said that perfect is the enemy of good, and our findings suggest that we can focus on the latter in terms of ART adherence, which is a much more appealing and sustainable message for the millions of people taking lifelong ART.

## COMPETING INTERESTS

JEH has been a consultant for Merck. All other authors declare no conflict of interest.

## AUTHORS’ CONTRIBUTIONS

ZaW drafted the initial manuscript. ZeW, CS, YK, MO, JEH and SL reviewed and edited the manuscript. ZeW conducted all analyses under the guidance of ZaW and SL. SL designed the original BEST study and led all study activities.

## FUNDING

This work is funded by the National Institute of Mental Health (Grant: R01MH110350, PI: Linnemayr).

## Supporting information


**Figure S1** Distribution of adherence by regimen
**Figure S2** Flow diagram of study participants
**Table S1**. Association between DTG and average adherence in the past 3 months leading up to the test
**Table S2**. Adherence and viral suppression under DTG versus non‐DTG ART regimens (1 month look‐back)
**Table S3**. Adherence interruptions and viral suppression under DTG versus non‐DTG ART (1‐month look‐back)
**Table S4**. Association between DTG and viral suppression (VS) (1‐month look‐back)
**Table S5**. Adherence and viral suppression under DTG versus non‐DTG ART regimens (6‐month look‐back)
**Table S6**. Adherence interruptions and viral suppression under DTG versus non‐DTG ART (6‐month look‐back)
**Table S7**. Association between DTG and viral suppression (VS) (6‐month look‐back)
**Table S8**. Adherence and viral suppression under DTG versus non‐DTG ART regimens (50 copies/ml)
**Table S9**. Adherence interruptions and viral suppression under DTG versus non‐DTG ART (50 copies/ml)
**Table S10**. Association between DTG and viral suppression (VS) (50 copies/ml)
**Table S11**. Adherence and viral suppression under DTG versus non‐DTG ART regimens (controlling for VS status in the previous test)
**Table S12**. Adherence interruptions and viral suppression under DTG versus non‐DTG ART (controlling for VS status in the previous test)
**Table S13**. List of most common regimens before and after switching to dolutagravir
**Table S14**. Adherence and viral suppression under DTG versus non‐DTG ART regimens logistics regression results
STROBE Statement—a checklist of items that should be included in reports of observational studies.

## Data Availability

Study data will not be publicly available due to IRB restrictions. Data can be made available to interested parties only by completing a registration and review process through the Mildmay Uganda IRB. Requests for access to the data should be sent to the corresponding author (wagnerru@usc.edu).
