# Supplementary material for: The association between adherence to antiretroviral therapy and viral suppression under dolutegravir‐based regimens: an observational cohort study from Uganda
Source: J Int AIDS Soc. 2024 Aug 18;27(8):e26350. doi: 10.1002/jia2.26350 (PMC11330848; doi:10.1002/jia2.26350)
Supplement: Supplementary file 1 — Figure S1 Distribution of adherence by regimen Figure S2 Flow diagram of study participants Table S1. Association between DTG and average adherence in the past 3 months leading up to the test Table S2. Adherence and viral suppression under DTG versus non‐DTG ART regimens (1 month look‐back) Table S3. Adherence interruptions and viral suppression under DTG versus non‐DTG ART (1‐month look‐back) Table S4. Association between DTG and viral suppression (VS) (1‐month look‐back) Table S5. Adherence and viral suppression under DTG versus non‐DTG ART regimens (6‐month look‐back) Table S6. Adherence interruptions and viral suppression under DTG versus non‐DTG ART (6‐month look‐back) Table S7. Association between DTG and viral suppression (VS) (6‐month look‐back) Table S8. Adherence and viral suppression under DTG versus non‐DTG ART regimens (50 copies/ml) Table S9. Adherence interruptions and viral suppression under DTG versus non‐DTG ART (50 copies/ml) Table S10. Association between DTG and viral suppression (VS) (50 copies/ml) Table S11. Adherence and viral suppression under DTG versus non‐DTG ART regimens (controlling for VS status in the previous test) Table S12. Adherence interruptions and viral suppression under DTG versus non‐DTG ART (controlling for VS status in the previous test) Table S13. List of most common regimens before and after switching to dolutagravir Table S14. Adherence and viral suppression under DTG versus non‐DTG ART regimens logistics regression results STROBE Statement—a checklist of items that should be included in reports of observational studies. [file JIA2-27-e26350-s001.docx]

**The Association Between Adherence to Antiretroviral Therapy and Viral Suppression Under Dolutegravir-Based Regimens: An Observational Cohort Study from Uganda**

[Supplementary Appendix]

Zachary Wagner, Zetianyu Wang, Chad Stecher, Yvonne Karamagi, Mary Odiit, Jessica E. Haberer, Sebastian Linnemayr

[Appendix Figures 3](#_Toc165634136)

[Figure S1 Distribution of adherence by regimen 3](#_Toc165634137)

[Figure S2 Flow diagram of study participants 4](#_Toc165634138)

[Appendix Tables 5](#_Toc165634139)

[Table S1. Association between DTG and average adherence in past 3 months leading up to the test 5](#_Toc165634140)

[Table S2. Adherence and viral suppression under DTG vs. non-DTG ART regimens (1 month look-back) 6](#_Toc165634141)

[Table S3. Adherence interruptions and viral suppression under DTG vs. non-DTG ART (1-month look-back) 7](#_Toc165634142)

[Table S4. Association between DTG and viral suppression (VS) (1-month look-back) 8](#_Toc165634143)

[Table S5. Adherence and viral suppression under DTG vs. non-DTG ART regimens (6-month look-back) 9](#_Toc165634144)

[Table S6. Adherence interruptions and viral suppression under DTG vs. non-DTG ART (6-month look-back) 10](#_Toc165634145)

[Table S7. Association between DTG and viral suppression (VS) (6-month look-back) 11](#_Toc165634146)

[Table S8. Adherence and viral suppression under DTG vs. non-DTG ART regimens (50 copies/mL) 12](#_Toc165634147)

[Table S9. Adherence interruptions and viral suppression under DTG vs. non-DTG ART (50 copies/mL) 13](#_Toc165634148)

[Table S10. Association between DTG and viral suppression (VS) (50 copies/mL) 14](#_Toc165634149)

[Table S11. Adherence and viral suppression under DTG vs. non-DTG ART regimens (controlling for VS status in the previous test) 15](#_Toc165634150)

[Table S12. Adherence interruptions and viral suppression under DTG vs. non-DTG ART (controlling for VS status in the previous test) 16](#_Toc165634151)

[Table S13. List of Most Common Regimens Before and After Switching to Dolutagravir 17](#_Toc165634152)

[Table S14. Adherence and viral suppression under DTG vs. non-DTG ART regimens logistics regression results 18](#_Toc165634153)

[STROBE Statement—checklist of items that should be included in reports of observational studies 19](#_Toc165634154)

# Appendix Figures

## Figure S1 Distribution of adherence by regimen


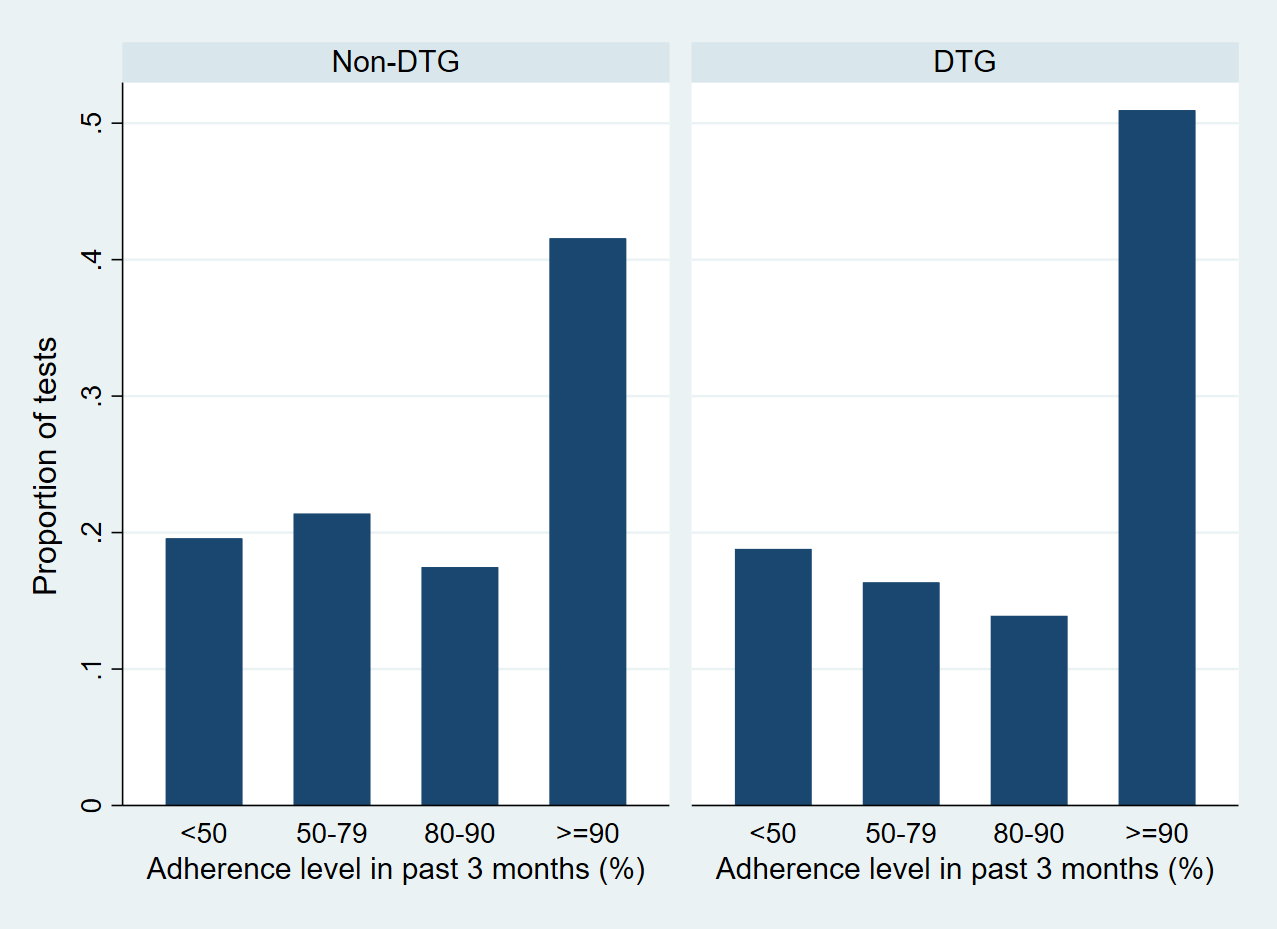


## Figure S2 Flow diagram of study participants

**
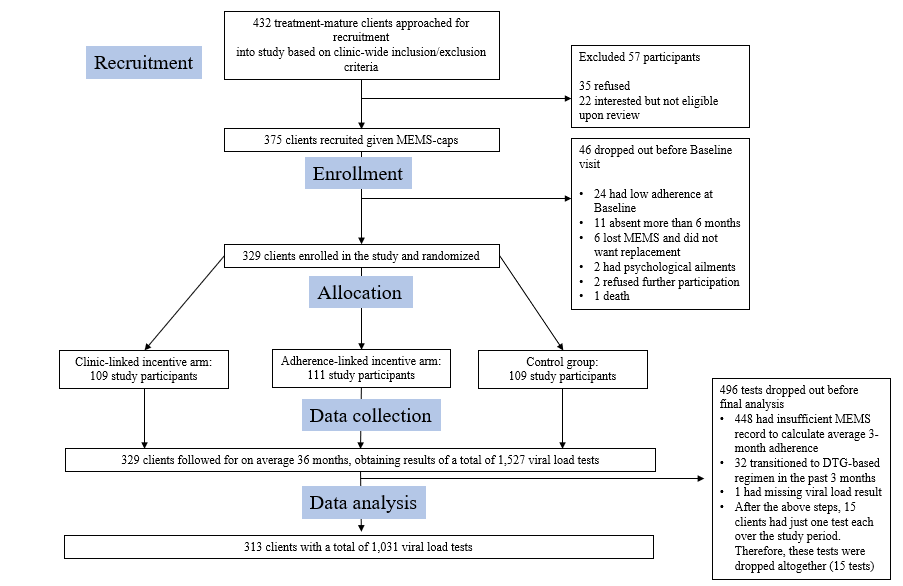
**

# Appendix Tables

| Table S1. Association between DTG and average adherence in past 3 months leading up to the test | | |
| --- | --- | --- |
|  | (1) | (2) |
|  | Unadjusted | Adjusted |
|  |  |  |
| On DTG | 0.03* | -0.08*** |
|  | (0.02) | (0.02) |
| Constant | 0.75*** | 0.79*** |
|  | (0.02) | (0.01) |
|  |  |  |
| Observations | 1,031 | 1,031 |
| R-squared | 0.00 | 0.71 |
| Number of Individuals | 313 | 313 |
| Number of Quarters | 15 | 15 |
| Individual FE | No | Yes |
| Quarter FE | No | Yes |
| Note: Analyses were conducted at test level. *** p<0.01, ** p<0.05, * p<0.1. Robust standard errors in parentheses. | | |

| Table S2. Adherence and viral suppression under DTG vs. non-DTG ART regimens (1 month look-back) | | | | | | | | |
| --- | --- | --- | --- | --- | --- | --- | --- | --- |
|  | Difference in Viral Suppression Relative to >90% | | | | | | | |
|  | non-DTG | | | | DTG | | | |
|  | Unadjusted  (95% CI) | p-value | Adjusted  (95% CI) | p-value | Unadjusted  (95% CI) | p-value | Adjusted  (95% CI) | p-value |
| 0-49% | -0.34 | 0.00 | -0.24 | 0.00 | -0.02 | 0.46 | 0.00 | 0.83 |
|  | (-0.46, -0.22) |  | (-0.37, -0.12) |  | (-0.08, 0.03) |  | (-0.07, 0.09) |  |
| 50-79% | -0.05 | 0.22 | -0.04 | 0.36 | 0.00 | 0.92 | 0.03 | 0.43 |
|  | (-0.13, 0.03) |  | (-0.13, 0.04) |  | (-0.06, 0.06) |  | (-0.05, 0.12) |  |
| 80-89% | -0.05 | 0.14 | -0.07 | 0.06 | 0.02 | 0.23 | 0.00 | 0.88 |
|  | (-0.13, 0.02) |  | (-0.15, 0.00) |  | (-0.01, 0.06) |  | (-0.05, 0.06) |  |
| Analyses were run at viral load test level. Adjusted models include individual fixed-effects and quarter-fixed effects (number of quarters = 15). Both unadjusted and adjusted models include 313 individuals. CIs and p-values were estimated with standard errors clustered by individual. | | | | | | | | |

| Table S3. Adherence interruptions and viral suppression under DTG vs. non-DTG ART (1-month look-back) | | | | | | | | |
| --- | --- | --- | --- | --- | --- | --- | --- | --- |
|  | Difference in Viral Suppression Relative to no gaps | | | | | | | |
|  | non-DTG | | | | DTG | | | |
| Duration of interruption | Unadjusted  (95% CI) | p-value | Adjusted  (95% CI) | p-value | Unadjusted  (95% CI) | p-value | Adjusted  (95% CI) | p-value |
| 1 day | -0.02 | 0.49 | 0.00 | 0.98 | 0.00 | 0.71 | -0.00 | 0.89 |
|  | (-0.09, 0.04) |  | (-0.06, 0.06) |  | (-0.03, 0.04) |  | (-0.05, 0.05) |  |
| 2 days | -0.08 | 0.06 | -0.02 | 0.52 | -0.04 | 0.13 | -0.00 | 0.88 |
|  | (-0.17, 0.00) |  | (-0.11, 0.05) |  | (-0.09, 0.01) |  | (-0.07, 0.06) |  |
| 3-5 days | -0.00 | 0.86 | -0.01 | 0.79 | -0.02 | 0.56 | 0.03 | 0.59 |
|  | (-0.10, 0.08) |  | (-0.09, 0.07) |  | (-0.09, 0.05) |  | (-0.09, 0.15) |  |
| 6 days and more | -0.34 | 0 | -0.24 | 0.00 | -0.02 | 0.64 | -0.01 | 0.75 |
|  | (-0.47, -0.21) |  | (-0.37, -0.11) |  | (-0.10, 0.06) |  | (-0.10, 0.07) |  |
| Analyses were run at viral load test level. Adjusted models include individual fixed-effects and quarter-fixed effects (number of quarters = 15). Both unadjusted and adjusted models include 313 individuals. 95% CIs and p-values were estimated with standard errors clustered by individual. The duration of interruption categories are not mutually exclusive. Therefore, the coefficients shown above are net effects of experiencing adherence gap of certain length, controlling for the presence of gaps of all other lengths. | | | | | | | | |

| Table S4. Association between DTG and viral suppression (VS) (1-month look-back) | | | | | | |
| --- | --- | --- | --- | --- | --- | --- |
|  | Difference in Viral Suppression for DTG compared to non-DTG ART | | | | | |
| Adherence Level | Share of VS  non-DTG | Share of VS  DTG | Unadjusted  (95% CI) | p-value | Adjusted  (95% CI) | p-value |
| All Levels of Adherence Pooled | 0.77 | 0.96 | 0.18 | 0.00 | 0.08 | 0.00 |
|  | (0.74, 0.80) | (0.94, 0.98) | (0.14, 0.23) |  | (0.02, 0.14) |  |
| 0-49% | 0.51 | 0.94 | 0.42 | 0.00 | 0.34 | 0.03 |
|  | (0.43, 0.60) | (0.88, 0.99) | (0.30, 0.54) |  | (0.01, 0.66) |  |
| 50-79% | 0.80 | 0.96 | 0.16 | 0.00 | -0.06 | 0.49 |
|  | (0.73, 0.86) | (0.92, 1.0) | (0.06, 0.26) |  | (-0.23, 0.11) |  |
| 80-89% | 0.79 | 0.98 | 0.18 | 0.00 | 0.15 | 0.08 |
|  | (0.73, 0.86) | (0.95, 1.0) | (0.10, 0.26) |  | (-0.02, 0.33) |  |
| >90% | 0.85 | 0.96 | 0.10 | 0.00 | -0.00 | 0.97 |
|  | (0.81, 0.89) | (0.93, 0.99) | (0.05, 0.15) |  | (-0.07, 0.07) |  |
| Analyses were run at viral load test level. Adjusted models include individual fixed-effects and quarter-fixed effects (number of quarters = 15). Both unadjusted and adjusted models include 313 individuals. 95% CIs and p-values were estimated with standard errors clustered by individual. Adherence level of 50-59%, 60-69%, and 70-79% were pooled together to gain sufficient observations to run TWFE regressions. | | | | | | |

| Table S5. Adherence and viral suppression under DTG vs. non-DTG ART regimens (6-month look-back) | | | | | | | | |
| --- | --- | --- | --- | --- | --- | --- | --- | --- |
|  | Difference in Viral Suppression Relative to >90% | | | | | | | |
|  | non-DTG | | | | DTG | | | |
|  | Unadjusted  (95% CI) | p-value | Adjusted  (95% CI) | p-value | Unadjusted  (95% CI) | p-value | Adjusted  (95% CI) | p-value |
| 0-49% | -0.31 | 0 | -0.06 | 0.42 | -0.01 | 0.67 | 0.01 | 0.78 |
|  | (-0.44, -0.18) |  | (-0.23, 0.09) |  | (-0.07, 0.04) |  | (-0.08, 0.11) |  |
| 50-79% | -0.07 | 0.09 | -0.11 | 0.05 | 0.00 | 0.90 | 0.04 | 0.41 |
|  | (-0.17, 0.01) |  | (-0.22, 0.00) |  | (-0.06, 0.07) |  | (-0.06, 0.14) |  |
| 80-89% | -0.03 | 0.53 | -0.05 | 0.24 | 0.03 | 0.01 | 0.01 | 0.61 |
|  | (-0.13, 0.07) |  | (-0.15, 0.03) |  | (0.00, 0.06) |  | (-0.05, 0.09) |  |
| Analyses were run at viral load test level. Adjusted models include individual fixed-effects and quarter-fixed effects (number of quarters = 15). Both unadjusted and adjusted models include 292 individuals. 95% CIs and p-values were estimated with standard errors clustered by individual. | | | | | | | | |

| Table S6. Adherence interruptions and viral suppression under DTG vs. non-DTG ART (6-month look-back) | | | | | | | | |
| --- | --- | --- | --- | --- | --- | --- | --- | --- |
|  | Difference in Viral Suppression Relative to no gaps | | | | | | | |
|  | non-DTG | | | | DTG | | | |
| Duration of interruption | Unadjusted  (95% CI) | p-value | Adjusted  (95% CI) | p-value | Unadjusted  (95% CI) | p-value | Adjusted  (95% CI) | p-value |
| 1 day | 0.03 | 0.58 | 0.00 | 0.92 | 0.05 | 0.24 | 0.07 | 0.28 |
|  | (-0.07, 0.14) |  | (-0.09, 0.10) |  | (-0.03, 0.14) |  | (-0.06, 0.21) |  |
| 2 days | -0.10 | 0.02 | -0.09 | 0.00 | -0.04 | 0.13 | -0.05 | 0.17 |
|  | (-0.18, -0.01) |  | (-0.16, -0.03) |  | (-0.10, 0.01) |  | (-0.13, 0.02) |  |
| 3-5 days | -0.04 | 0.33 | -0.02 | 0.49 | 0.00 | 0.99 | 0.02 | 0.61 |
|  | (-0.12, 0.04) |  | (-0.11, 0.05) |  | (-0.07, 0.07) |  | (-0.06, 0.11) |  |
| 6 days and more | -0.18 | 0.00 | -0.08 | 0.04 | 0.02 | 0.44 | 0.05 | 0.22 |
|  | (-0.27, -0.09) |  | (-0.15, -0.00) |  | (-0.03, 0.08) |  | (-0.03, 0.13) |  |
| Analyses were run at viral load test level. Adjusted models include individual fixed-effects and quarter-fixed effects (number of quarters = 15). Both unadjusted and adjusted models include 292 individuals. 95% CIs and p-values were estimated with standard errors clustered by individual. The duration of interruption categories are not mutually exclusive. Therefore, the coefficients shown above are net effects of experiencing adherence gap of certain length, controlling for the presence of gaps of all other lengths. | | | | | | | | |

| Table S7. Association between DTG and viral suppression (VS) (6-month look-back) | | | | | | |
| --- | --- | --- | --- | --- | --- | --- |
|  | Difference in Viral Suppression for DTG compared to non-DTG ART | | | | | |
| Adherence Level | Share of VS  non-DTG | Share of VS  DTG | Unadjusted  (95% CI) | p-value | Adjusted  (95% CI) | p-value |
| All Levels of Adherence Pooled | 0.78 | 0.96 | 0.18 | 0.00 | 0.02 | 0.36 |
|  | (0.74, 0.81) | (0.94, 0.98) | (0.13, 0.23) |  | (-0.03, 0.09) |  |
| 0-49% | 0.55 | 0.94 | 0.39 | 0.00 | 0.07 | 0.66 |
|  | (0.46, 0.64) | (0.89, 1.0) | (0.26, 0.52) |  | (-0.28, 0.43) |  |
| 50-79% | 0.78 | 0.96 | 0.17 | 0.00 | 0.08 | 0.68 |
|  | (0.71, 0.86) | (0.91, 1.0) | (0.06, 0.28) |  | (-0.32, 0.49) |  |
| 80-89% | 0.83 | 1 | 0.16 | 0.00 | 0.03 | 0.75 |
|  | (0.76, 0.90) | NA | (0.06, 0.26) |  | (-0.17, 0.23) |  |
| >90% | 0.86 | 0.96 | 0.09 | 0.00 | -0.00 | 0.94 |
|  | (0.82, 0.91) | (0.93, 0.99) | (0.03, 0.15) |  | (-0.08, 0.07) | (0.82, 0.91) |
| Analyses were run at viral load test level. Adjusted models include individual fixed-effects and quarter-fixed effects (number of quarters = 15). Both unadjusted and adjusted models include 292 individuals. 95% CIs and p-values were estimated with standard errors clustered by individual. 95% CIs and p-values were estimated with standard errors clustered by individual. Adherence level of 50-59%, 60-69%, and 70-79% were pooled together to gain sufficient observations to run TWFE regressions. | | | | | | |

| Table S8. Adherence and viral suppression under DTG vs. non-DTG ART regimens (50 copies/mL) | | | | | | | | |
| --- | --- | --- | --- | --- | --- | --- | --- | --- |
|  | Difference in Viral Suppression Relative to >90% | | | | | | | |
|  | non-DTG | | | | DTG | | | |
|  | Unadjusted  (95% CI) | p-value | Adjusted  (95% CI) | p-value | Unadjusted  (95% CI) | p-value | Adjusted  (95% CI) | p-value |
| 0-49% | -0.34 | 0 | -0.22 | 0.00 | 0.04 | 0.36 | 0.17 | 0.01 |
|  | (-0.46, -0.21) |  | (-0.38, -0.05) |  | (-0.04, 0.13) |  | (0.03, 0.31) |  |
| 50-79% | -0.14 | 0.01 | -0.14 | 0.03 | -0.03 | 0.52 | 0.04 | 0.50 |
|  | (-0.25, -0.03) |  | (-0.27, -0.01) |  | (-0.15, 0.08) |  | (-0.09, 0.19) |  |
| 80-89% | -0.01 | 0.75 | 0.03 | 0.54 | 0.02 | 0.63 | 0.05 | 0.46 |
|  | (-0.11, 0.08) |  | (-0.06, 0.12) |  | (-0.08, 0.13) |  | (-0.08, 0.19) |  |
| Analyses were run at viral load test level. Adjusted models include individual fixed-effects and quarter-fixed effects (number of quarters = 15). Both unadjusted and adjusted models include 313 individuals. 95% CIs and p-values were estimated with standard errors clustered by individual. | | | | | | | | |

| Table S9. Adherence interruptions and viral suppression under DTG vs. non-DTG ART (50 copies/mL) | | | | | | | | |
| --- | --- | --- | --- | --- | --- | --- | --- | --- |
|  | Difference in Viral Suppression Relative to no gaps | | | | | | | |
|  | non-DTG | | | | DTG | | | |
| Duration of interruption | Unadjusted  (95% CI) | p-value | Adjusted  (95% CI) | p-value | Unadjusted  (95% CI) | p-value | Adjusted  (95% CI) | p-value |
| 1 day | -0.02 | 0.58 | 0.00 | 0.91 | 0.02 | 0.57 | 0.09 | 0.16 |
|  | (-0.12, 0.07) |  | (-0.09, 0.11) |  | (-0.07, 0.12) |  | (-0.03, 0.22) |  |
| 2 days | -0.09 | 0.05 | -0.05 | 0.20 | -0.04 | 0.27 | -0.01 | 0.75 |
|  | (-0.18, 0.00) |  | (-0.15, 0.03) |  | (-0.13, 0.03) |  | (-0.13, 0.09) |  |
| 3-5 days | -0.02 | 0.62 | -0.05 | 0.22 | -0.03 | 0.52 | 0.00 | 0.95 |
|  | (-0.10, 0.06) |  | (-0.14, 0.03) |  | (-0.14, 0.07) |  | (-0.13, 0.14) |  |
| 6 days and more | -0.26 | 0 | -0.16 | 0.00 | 0.05 | 0.30 | 0.01 | 0.80 |
|  | (-0.36, -0.16) |  | (-0.27, -0.06) |  | (-0.04, 0.14) |  | (-0.10, 0.14) |  |
| Analyses were run at viral load test level. Adjusted models include individual fixed-effects and quarter-fixed effects (number of quarters = 15). Both unadjusted and adjusted models include 313 individuals. 95% CIs and p-values were estimated with standard errors clustered by individual. The duration of interruption categories are not mutually exclusive. Therefore, the coefficients shown above are net effects of experiencing adherence gap of certain length, controlling for the presence of gaps of all other lengths. | | | | | | | | |

| Table S10. Association between DTG and viral suppression (VS) (50 copies/mL) | | | | | | |
| --- | --- | --- | --- | --- | --- | --- |
|  | Difference in Viral Suppression for DTG compared to non-DTG ART | | | | | |
| Adherence Level | Share of VS  non-DTG | Share of VS  DTG | Unadjusted  (95% CI) | p-value | Adjusted  (95% CI) | p-value |
| All Levels of Adherence Pooled | 0.68 | 0.86 | 0.17 | 0.00 | 0.05 | 0.21 |
|  | (0.64, 0.71) | (0.82, 0.89) | (0.11, 0.24) |  | (-0.03, 0.15) |  |
| 0-49% | 0.43 | 0.89 | 0.46 | 0.00 | 0.08 | 0.66 |
|  | (0.35, 0.52) | (0.82, 0.97) | (0.32, 0.59) |  | (-0.32, 0.50) |  |
| 50-79% | 0.64 | 0.81 | 0.17 | 0.02 | 0.04 | 0.83 |
|  | (0.56, 0.72) | (0.71, 0.91) | (0.02, 0.32) |  | (-0.41, 0.51) |  |
| 80-89% | 0.76 | 0.88 | 0.11 | 0.08 | 0.26 | 0.03 |
|  | (0.68, 0.84) | (0.79, 0.97) | (-0.01, 0.24) |  | (0.02, 0.51) |  |
| >90% | 0.78 | 0.85 | 0.07 | 0.06 | -0.00 | 0.92 |
| Analyses were run at viral load test level. Adjusted models include individual fixed-effects and quarter-fixed effects (number of quarters = 15). Both unadjusted and adjusted models include 313 individuals. 95% CIs and p-values were estimated with standard errors clustered by individual. Adherence level of 50-59%, 60-69%, and 70-79% were pooled together to gain sufficient observations to run TWFE regressions. | | | | | | |

| Table S11. Adherence and viral suppression under DTG vs. non-DTG ART regimens (controlling for VS status in the previous test) | | | | | | | | |
| --- | --- | --- | --- | --- | --- | --- | --- | --- |
|  | Difference in Viral Suppression Relative to >90% | | | | | | | |
|  | non-DTG | | | | DTG | | | |
|  | Unadjusted  (95% CI) | p-value | Adjusted  (95% CI) | p-value | Unadjusted  (95% CI) | p-value | Adjusted  (95% CI) | p-value |
| 0-49% | -0.21 | 0.00 | -0.15 | 0.03 | 0.01 | 0.60 | 0.07 | 0.12 |
|  | (-0.31, -0.11) |  | (-0.29, -0.01) |  | (-0.03, 0.06) |  | (-0.02, 0.17) |  |
| 50-79% | -0.08 | 0.02 | -0.10 | 0.11 | 0.01 | 0.55 | 0.08 | 0.05 |
|  | (-0.16, -0.00) |  | (-0.22, 0.02) |  | (-0.04, 0.08) |  | (-0.00, 0.17) |  |
| 80-89% | 0.01 | 0.57 | 0.03 | 0.32 | 0.00 | 0.73 | 0.01 | 0.79 |
|  | (-0.04, 0.07) |  | (-0.03, 0.11) |  | (-0.04, 0.05) |  | (-0.07, 0.09) |  |
| Analyses were run at viral load test level. VS status in the immediate prior test was included as a control variable in each of the above models. Both unadjusted and adjusted models include 313 individuals. 95% CIs and p-values were estimated with standard errors clustered by individual. | | | | | | | | |

| Table S12. Adherence interruptions and viral suppression under DTG vs. non-DTG ART (controlling for VS status in the previous test) | | | | | | | | |
| --- | --- | --- | --- | --- | --- | --- | --- | --- |
|  | Difference in Viral Suppression Relative to no gaps | | | | | | | |
|  | non-DTG | | | | DTG | | | |
| Duration of interruption | Unadjusted  (95% CI) | p-value | Adjusted  (95% CI) | p-value | Unadjusted  (95% CI) | p-value | Adjusted  (95% CI) | p-value |
| 1 day | -0.02 | 0.51 | -0.03 | 0.46 | 0.05 | 0.08 | 0.06 | 0.09 |
|  | (-0.09, 0.04) |  | (-0.11, 0.05) |  | (-0.00, 0.11) |  | (-0.01, 0.14) |  |
| 2 days | -0.05 | 0.15 | -0.03 | 0.47 | -0.03 | 0.19 | -0.03 | 0.44 |
|  | (-0.13, 0.02) |  | (-0.11, 0.05) |  | (-0.08, 0.01) |  | (-0.11, 0.04) |  |
| 3-5 days | -0.00 | 0.95 | -0.04 | 0.26 | -0.01 | 0.56 | 0.00 | 0.95 |
|  | (-0.06, 0.06) |  | (-0.12, 0.03) |  | (-0.08, 0.04) |  | (-0.09, 0.09) |  |
| 6 days and more | -0.20 | 0 | -0.13 | 0.00 | 0.02 | 0.44 | 0.02 | 0.54 |
|  | (-0.28, -0.12) |  | (-0.22, -0.04) |  | (-0.03, 0.07) |  | (-0.06, 0.11) |  |
| Analyses were run at viral load test level. VS status in the immediate prior test was included as a control variable in each of the above models. Both unadjusted and adjusted models include 313 individuals. 95% CIs and p-values were estimated with standard errors clustered by individual. The duration of interruption categories is not mutually exclusive. Therefore, the coefficients shown above are net effects of experiencing adherence gap of certain length, controlling for the presence of gaps of all other lengths. | | | | | | | | |

| Table S13. List of Most Common Regimens Before and After Switching to Dolutagravir | | |
| --- | --- | --- |
|  | Number of daily tabs | N (% of study participants) |
| Initial regimens |  |  |
| Tenofovir/Lamivudine/Efavirenz | 1 | 88 (29.1%) |
| Tenofovir/Lamivudine/Atazanavir/Ritonvir | 2 | 81 (26.7%) |
| Zidovidine/Lamivudine/Efavirenz | 3 | 29 (9.7%) |
| Zidovidine/Lamivudine/Nevirapine | 2 | 28 (9.1%) |
| Zidovidine/Lamivudine/Atazanavir/Ritonvir | 3 | 27 (8.8%) |
| Tenofovir/Lamivudine/Lopinavir/Ritonavir | 5 | 20 (6.7%) |
| Tenofovir/Lamivudine/Nevirapine | 3 | 17 (5.8%) |
| Other |  | 13 (4.2%) |
| Regimens after switch to DTG |  |  |
| Tenofovir/Lamivudine/Dolutagravir | 1 | 249 (82.2%) |
| Zidovidine/Lamivudine/Dolutegravir | 3 | 35 (11.6%) |
| Abacavir/Lamivudine/Dolutegravir | 1 | 6 (2.0%) |
| Darunavir/Ritonavir/Dolutegravir | 5 | 3 (1.0%) |
| Other |  | 10 (3.3%) |
| Note: 27 study participants who never received DTG-based regimen during our study period were excluded from this table. Regimens received by less than 1% of the study participants were grouped into the “Other” category. | | |

| Table S14. Adherence and viral suppression under DTG vs. non-DTG ART regimens logistics regression results | | | | | | | | |
| --- | --- | --- | --- | --- | --- | --- | --- | --- |
|  | Association with Viral Suppression | | | | | | | |
|  | non-DTG | | | | DTG | | | |
| Adherence Level: Relative to >90% | Unadjusted | p-value | Adjusted | p-value | Unadjusted | p-value | Adjusted | p-value |
| 0-49% | -0.32 | 0.00 | NA | NA | -0.00 | 0.82 | NA | NA |
| 50-79% | -0.12 | 0.00 | NA | NA | -0.01 | 0.73 | NA | NA |
| 80-89% | 0.00 | 0.81 | NA | NA | 0.02 | 0.45 | NA | NA |

Note: 1) unadjusted effect sizes were generated by first estimating the predicted likelihood of viral suppression for each (adherence level , regimen) combination and then test whether the differences between these predicted values are significantly different from zero. For example, the effect size of 0-49% adherence level under non-DTG regimen (-0.32) was estimated by taking the difference in predicted likelihood of VS for 0-49% adherence level under non-DTG (0.54, not shown in the table) and that for above 90% adherence (the reference category) under non-DTG (0.86, not shown in the table). A chi-square test was performed to assess whether this difference was significantly different from zero. 2) Adjusted effect sizes were not estimated because fixed effects estimators under nonlinear models such as logistic models suffer from potential issues such as incidental parameter problem (Neyman and Scott, 1948).

*Reference for this table:*

Neyman, Jerzy, and Elizabeth L. Scott. "Consistent estimates based on partially consistent observations." Econometrica: journal of the Econometric Society (1948): 1-32.

# STROBE Statement—checklist of items that should be included in reports of observational studies

|  | Item No | Recommendation | Page  No |
| --- | --- | --- | --- |
| **Title and abstract** | 1 | (*a*) Indicate the study’s design with a commonly used term in the title or the abstract | 1 |
|  |  | (*b*) Provide in the abstract an informative and balanced summary of what was done and what was found | 2 |
| Introduction | | | |
| Background/rationale | 2 | Explain the scientific background and rationale for the investigation being reported | 3 |
| Objectives | 3 | State specific objectives, including any prespecified hypotheses | 4 |
| Methods | | | |
| Study design | 4 | Present key elements of study design early in the paper | 4 |
| Setting | 5 | Describe the setting, locations, and relevant dates, including periods of recruitment, exposure, follow-up, and data collection | 4 |
| Participants | 6 | (*a*) *Cohort study*—Give the eligibility criteria, and the sources and methods of selection of participants. Describe methods of follow-up  *Case-control study*—Give the eligibility criteria, and the sources and methods of case ascertainment and control selection. Give the rationale for the choice of cases and controls  *Cross-sectional study*—Give the eligibility criteria, and the sources and methods of selection of participants | 5 |
|  |  | (*b*) *Cohort study*—For matched studies, give matching criteria and number of exposed and unexposed  *Case-control study*—For matched studies, give matching criteria and the number of controls per case | NA (not a matched study) |
| Variables | 7 | Clearly define all outcomes, exposures, predictors, potential confounders, and effect modifiers. Give diagnostic criteria, if applicable | 5,6 |
| Data sources/ measurement | 8* | For each variable of interest, give sources of data and details of methods of assessment (measurement). Describe comparability of assessment methods if there is more than one group | *5,6* |
| Bias | 9 | Describe any efforts to address potential sources of bias | 6,7 |
| Study size | 10 | Explain how the study size was arrived at | 5 |
| Quantitative variables | 11 | Explain how quantitative variables were handled in the analyses. If applicable, describe which groupings were chosen and why | 6,7 |
| Statistical methods | 12 | (*a*) Describe all statistical methods, including those used to control for confounding | 6,7 |
|  |  | (*b*) Describe any methods used to examine subgroups and interactions | 6,7 |
|  |  | (*c*) Explain how missing data were addressed | NA (no missing data) |
|  |  | (*d*) *Cohort study*—If applicable, explain how loss to follow-up was addressed  *Case-control study*—If applicable, explain how matching of cases and controls was addressed  *Cross-sectional study*—If applicable, describe analytical methods taking account of sampling strategy | NA |
|  |  | (*e*) Describe any sensitivity analyses | 6 |

Continued on next page

| Results | | | |
| --- | --- | --- | --- |
| Participants | 13* | (a) Report numbers of individuals at each stage of study—eg numbers potentially eligible, examined for eligibility, confirmed eligible, included in the study, completing follow-up, and analysed | 7 |
|  |  | (b) Give reasons for non-participation at each stage | 7 |
|  |  | (c) Consider use of a flow diagram | Appendix |
| Descriptive data | 14* | (a) Give characteristics of study participants (eg demographic, clinical, social) and information on exposures and potential confounders | 7 |
|  |  | (b) Indicate number of participants with missing data for each variable of interest | 7 |
|  |  | (c) *Cohort study*—Summarise follow-up time (eg, average and total amount) | 5 |
| Outcome data | 15* | *Cohort study*—Report numbers of outcome events or summary measures over time | *8* |
|  |  | *Case-control study—*Report numbers in each exposure category, or summary measures of exposure | *NA* |
|  |  | *Cross-sectional study—*Report numbers of outcome events or summary measures | *NA* |
| Main results | 16 | (*a*) Give unadjusted estimates and, if applicable, confounder-adjusted estimates and their precision (eg, 95% confidence interval). Make clear which confounders were adjusted for and why they were included | 10,11,12 |
|  |  | (*b*) Report category boundaries when continuous variables were categorized | 10,11 |
|  |  | (*c*) If relevant, consider translating estimates of relative risk into absolute risk for a meaningful time period | NA |
| Other analyses | 17 | Report other analyses done—eg analyses of subgroups and interactions, and sensitivity analyses | 13 |
| Discussion | | | |
| Key results | 18 | Summarise key results with reference to study objectives | 14 |
| Limitations | 19 | Discuss limitations of the study, taking into account sources of potential bias or imprecision. Discuss both direction and magnitude of any potential bias | 16 |
| Interpretation | 20 | Give a cautious overall interpretation of results considering objectives, limitations, multiplicity of analyses, results from similar studies, and other relevant evidence | 15,16 |
| Generalisability | 21 | Discuss the generalisability (external validity) of the study results | 16 |
| Other information | | | |
| Funding | 22 | Give the source of funding and the role of the funders for the present study and, if applicable, for the original study on which the present article is based | 17 |

*Give information separately for cases and controls in case-control studies and, if applicable, for exposed and unexposed groups in cohort and cross-sectional studies.

**Note:** An Explanation and Elaboration article discusses each checklist item and gives methodological background and published examples of transparent reporting. The STROBE checklist is best used in conjunction with this article (freely available on the Web sites of PLoS Medicine at http://www.plosmedicine.org/, Annals of Internal Medicine at http://www.annals.org/, and Epidemiology at http://www.epidem.com/). Information on the STROBE Initiative is available at www.strobe-statement.org.
